# Age trumps metabolism: No independent association between lipids, statins, and prostate enlargement in a metabolically controlled cohort

**DOI:** 10.1016/j.clinsp.2026.100939

**Published:** 2026-05-20

**Authors:** Lucas Neves de Oliveira, Ricardo Brianezi Tiraboschi, Caroline Santos Silva, Jair Bomfim Santos, Mateus Neves de Oliveira, Cristiano Mendes Gomes, Jose Bessa Junior

**Affiliations:** a Department of Public Health, Universidade Estadual de Feira de Santana, Feira de Santana, BA, Brazil; b Division of Urology, Faculdade de Medicina da Universidade Estadual de Feira de Santana, Feira de Santana, BA, Brazil; c Department of Public Health, Escola Bahiana de Medicina e Saúde Pública, Salvador, BA, Brazil; d Division of Urology, Faculdade de Medicina, Universidade de São Paulo, São Paulo, SP, Brazil

**Keywords:** Prostate enlargement, Lipids, Statins, Metabolic syndrome, Aging, Risk factors, Benign prostatic hyperplasia (BPH), Cross-sectional study

## Abstract

**Objective:**

To evaluate the association between lipid profile, statin use, metabolic syndrome, and clinic. Prostate Enlargement (PE) in adult men attending a urology outpatient clinic.

**Methods:**

A cross-sectional study was conducted among 1117 men aged 40-years or older. Prostate volume was measured by ultrasonography; a volume ≥ 40 mL was considered the threshold for enlargement. Independent variables included lipid fractions, prescription-based statin use, and components of metabolic syndrome. Multivariate logistic regression models were applied, adjusted for age, hypertension, and medication exposure.

**Results:**

The median age was 63-years (IQR: 55–71), and the median prostate volume was 34.0 mL (IQR: 26.0–48.0). Statin use was identified in 36.5% of the sample, and the prevalence of PE was 38.9%. Age was the only factor independently associated with PE (OR = 1.07; 95% CI: 1.05–1.09; p < 0.001). Metabolic syndrome was associated with PE in crude analysis; however, this association lost statistical significance after adjustment for potential confounders.

**Conclusion:**

Age was the only independent determinant of prostate enlargement in this metabolically controlled cohort. Lipid parameters and prescription-based statin use showed no adjusted association with prostate volume; however, the lack of detailed statin exposure data warrants cautious interpretation of these findings.

## Highlights

• This cross-sectional study evaluated 1117 men aged ≥ 40-years and focused exclusively on anatomical prostate enlargement (PE ≥ 40 mL), explicitly distinguishing prostate morphology from symptomatic Benign Prostatic Hyperplasia (BPH).
• Age was the only variable independently associated with prostate enlargement, remaining statistically significant across multivariable models and all sensitivity analyses.
• Lipid fractions (HDL, LDL, triglycerides, and total cholesterol) and a prescription-based measure of statin use showed no independent association with prostate volume, including in analyses restricted to non-statin users, IPTW-weighted models, and LDL-stratified subgroups.
• Metabolic syndrome was associated with prostate enlargement in crude analyses, but this association was fully attenuated after age adjustment; correlation matrices and variance inflation factors confirmed the absence of problematic collinearity.
• In this metabolically well-controlled clinical cohort with a high prevalence of statin therapy, prostate enlargement appeared to be predominantly age-related rather than driven by modifiable metabolic or pharmacologic factors.

## Introduction

Benign Prostatic Hyperplasia (BPH) is one of the most common urological conditions among men over 50. It represents a clinical syndrome, traditionally associated with benign enlargement of the prostate's transition zone, here referred to as Prostate Enlargement (PE). Importantly, PE is an anatomical finding, whereas BPH is a symptomatic condition, and the two are not invariably concordant. This anatomical enlargement may lead to Lower Urinary Tract Symptoms (LUTS), significantly impacting quality of life, although prostate size alone does not define clinical BPH.^1^ It is estimated that over 50% of men between 60 and 69-years have clinically significant prostate enlargement, with prevalence exceeding 80% after the age of 70.^2^^,^^3^ The pathophysiology underlying prostate enlargement involves hormonal, inflammatory, genetic, and metabolic mechanisms, reflecting the complexity of prostatic growth during aging.^1^

Recent studies have suggested potential interactions between metabolic disorders and PE, particularly in Metabolic Syndrome (MetS). MetS is defined by the presence of at least three of the following: abdominal obesity, hypertension, dyslipidemia (reduced HDL and/or elevated triglycerides), and fasting hyperglycemia.^4^^,^^5^ Epidemiological evidence indicates that individuals with MetS are at greater risk of developing BPH, likely through mechanisms related to insulin resistance, chronic low-grade inflammation, and androgen axis alterations.^4^, ^5^, ^6^

Among the components of MetS, lipid alterations and their pharmacological control through statins have drawn special interest. Experimental studies have demonstrated that statins, in addition to lowering cholesterol, exhibit antiproliferative, anti-inflammatory, and mevalonate pathway-modulating effects, which may influence prostate growth.^6^ However, clinical results remain conflicting. While some studies report an association between dyslipidemia and prostate enlargement,^7^ others found no significant relationship, especially in populations with reasonable metabolic control.^8^^,^^9^

A commonly overlooked aspect in literature is the distinction between individuals with untreated dyslipidemia and those under effective pharmacological control. Statins tend to homogenize lipid levels, making it harder to detect clinically relevant gradients.^10^ Moreover, studies that fail to adjust for age, the primary determinant of prostatic growth, may be subject to residual confounding. Additionally, previous clinical studies have varied widely in their approaches ‒ some define PE differently, others assess different types or doses of statins, and many overlook important hormonal factors known to influence prostate growth.^8^, ^9^, ^10^

Given this scenario, the present study aims to evaluate the association between prostate volume and lipid profile variables, statin use, and metabolic syndrome in a clinical cohort of adult men. The outcome reflects anatomical PE (volume ≥40 mL), not clinical BPH, as Lower Urinary Tract Symptoms (LUTS) were not collected. Therefore, the findings apply to prostate morphology rather than symptomatic disease. The authors hypothesize that, in a population with widespread lipid control, these variables are not independently associated with prostate enlargement as an anatomical finding, while acknowledging that prostate volume does not necessarily translate into symptomatic BPH. This distinction underscores the importance of differentiating morphological changes from clinical manifestations when interpreting metabolic associations with prostatic growth.

## Methods

### Study design and population

This cross-sectional observational study involves 1117 adult men treated at a specialized urology outpatient clinic. Data collection was conducted between 2021 and 2023. Participants with available data on prostate volume, lipid profile, statin use, hypertension, and other metabolic variables were included. Patients with active prostate cancer, prior prostatectomy, or use of finasteride within the last six months were excluded.^5^ The cross-sectional design was chosen for its feasibility in evaluating clinical associations in large samples using routine data, although it does not allow causal inference.^11^ This study was conducted and reported in accordance with the STROBE (Strengthening the Reporting of Observational Studies in Epidemiology) guidelines.

The primary outcome was prostate enlargement, defined as a volume ≥ 40 mL measured via routine transabdominal ultrasound. This cutoff was based on studies using it as a morphological criterion for clinically significant hyperplasia.^5^ All ultrasound exams were performed by the same radiology team using a standardized protocol, minimizing measurement variability. Both image acquisition and prostate volume calculations were performed by the radiology team using the ellipsoid formula (height × width × length × 0.52) as part of routine clinical practice. The radiology team was not blinded to clinical or laboratory data, reflecting real-world practice in this outpatient setting. Although the examinations were standardized and conducted by the same team, the authors did not perform a formal repeatability assessment (e.g., inter- or intraobserver agreement metrics), which is a limitation inherent to the observational, real-world nature of this primary care-based study.

This study was approved by the Research Ethics Committee of the State University of Feira de Santana under protocol n° 97,443,018.3.0000.0053, position statement 3.057.301. All participants provided written informed consent. This research received no funding. The data that support the findings of this study are available from the corresponding author upon reasonable request.

### Independent variables included

Independent variables included components of Metabolic Syndrome (MetS) and MetS itself, defined according to NCEP ATP III criteria. Metabolic syndrome was diagnosed when three or more of the following criteria were met: 1) Waist circumference > 102 cm; 2) Triglycerides ≥ 150 mg/dL; 3) HDL cholesterol < 40 mg/dL; 4) Blood pressure ≥ 130 × 85 mmHg or antihypertensive medication use; and 5) Fasting glucose ≥ 100 mg/dL or use of antidiabetic medication. Lipid parameters (HDL, LDL, triglycerides, and total cholesterol) were measured after fasting using automated methods and expressed in milligrams per deciliter (mg/dL).^12^

Statin use was defined as a prescription-based measure obtained from electronic medical records at the time of data collection, without detailed information on type, potency, duration, or adherence.

### Statistical analysis

Continuous data were summarized as medians and Interquartile Ranges (IQRs). Categorical variables were expressed as absolute and relative frequencies.

The Mann-Whitney test was used to compare continuous variables between groups (with and without PE). Pearson's Chi-Square or Fisher's exact test was used for categorical variables, as appropriate. Spearman's coefficient was used to analyze the correlation between prostate volume and continuous variables.

To evaluate potential collinearity between age and metabolic variables, Pearson and Spearman correlation matrices were computed between age and LDL, HDL, triglycerides, glycemia, hypertension, waist circumference, and MetS. Variance Inflation Factors (VIFs) were also calculated for the complete multivariable model to confirm the absence of harmful collinearity.

Logistic regression analysis was performed to evaluate the association between prostate enlargement (≥ 40 mL) and clinical-metabolic variables. The model included age and metabolic syndrome as independent variables, given their clinical relevance and statistical significance in univariate analysis. Results were expressed as Odds Ratios (OR) with 95% Confidence Intervals (95% CI). A p-value < 0.05 was considered statistically significant.

Additional sensitivity analyses and an exploratory mediation analysis are reported in the Supplementary Appendix (Tables S1–S5).

All statistical analyses were performed using GraphPad Prism version 9.0 (GraphPad Software, San Diego, CA, USA) and MedCalc version 20.0 (MedCalc Software Ltd, Ostend, Belgium). Propensity score analyses (IPTW) were conducted in R version 4.4.2 using the car package, and mediation analyses were performed in R version 4.4.2 using the mediation package.

## Results

The study population consisted of 1117 adult men aged 40-years or older, with a median age of 63 (IQR: 55–71 years). Table 1 summarizes sociodemographic and clinical characteristics, including prostate volume, PSA, testosterone, lipid profile, hypertension, and statin use.

**Table 1:** Characteristics of the study sample.

Variable	Median (IQR) or n (%)
Age (years)	63 (55–71)
Prostate volume (mL)	34.0 (26.0–48.0)
Total PSA (ng/mL)	1.10 (0.61–2.04)
Total testosterone (ng/dL)	464 (351–618)
Triglycerides (mg/dL)	97 (78–121)
HDL cholesterol (mg/dL)	49 (41–57)
LDL cholesterol (mg/dL)	107 (88–128)
Total cholesterol (mg/dL)	182 (156–208)
Glycemia (mg/dL)	97 (90‒108)
Prostate Enlargement (≥ 40 mL)	435 (38.9%)
Hypertension	555 (49.7%)
WC > 102 cm	357 (31.9%)
Statin use	408 (36.5%)
Metabolic syndrome	330 (29.5%)

Table 2 summarizes univariate comparisons between individuals with and without Prostate Enlargement (PE). Patients with PE were significantly older and had a higher prevalence of metabolic syndrome. No significant differences were observed regarding isolated lipid profile parameters, statin use, obesity, glycemia, or hypertension.

**Table 2:** Comparison of clinical and metabolic variables between patients with and without PE.

	PE (≥ 40 mL) n = 435	No PE (< 40 mL) n = 682	p-value
**Age (years),**	67 (61–73)	58 (50–65)	***<0.001***
**Metabolic syndrome, n (%)**	150 (34.5%)	180 (26.4%)	***0.005***
**HDL cholesterol (mg/dL)**	48 (41–56)	50 (42–58)	0.083
**LDL cholesterol (mg/dL)**	106 (87–127)	108 (89–129)	0.462
**Triglycerides (mg/dL)**	96 (78–119)	98 (79–123)	0.493
**Glycemia (mg/dL)**	97 (90–109)	97 (90–107)	0.552
**Total cholesterol (mg/dL)**	182 (155–207)	182 (158–210)	0.692
**Statin use, n (%)**	163 (37.4%)	245 (35.9%)	0.949
**WC > 102cm n (%)**	152 (34.9%)	205 (30.1%)	0.010
**Hypertension, n (%)**	231 (53.1%)	324 (47.5%)	0.075

Values are presented as median (IQR) for continuous variables and n (%) for categorical variables. p-values were obtained using the Mann-Whitney *U test* for comparisons of continuous variables between groups and Pearson’s χ² test or Fisher’s exact test for categorical variables, as appropriate.

In the multivariate logistic regression analysis, only age showed a statistically significant association with prostate enlargement. Specifically, each year of age increased the odds of prostate enlargement by 7% (OR = 1.07 per-year; 95% CI: 1.05–1.09; p < 0.001). Statin use, ascertained from prescription records, hypertension, glycemia, obesity, and all lipid fractions, was not significantly associated with PE.

Sensitivity analyses (Tables S1–S4) and an exploratory mediation analysis (Table S5) yielded results consistent with the primary model and are reported in the Supplementary Appendix.

**Fig. 1:**
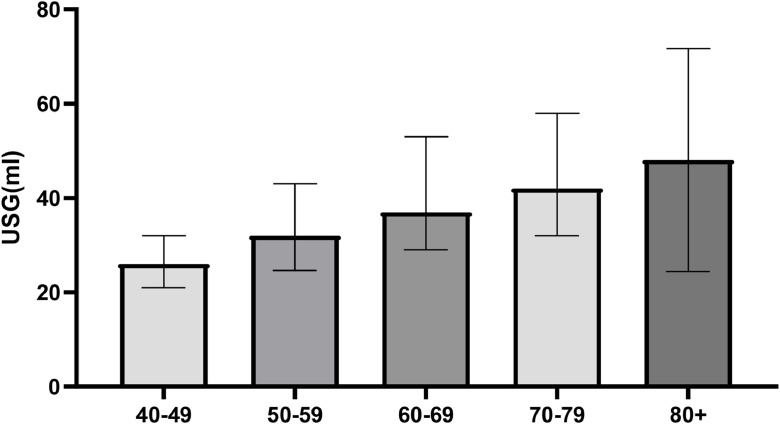
Median prostate volume (mL) by age group, measured by transabdominal ultrasound. Bars represent median values, and whiskers interquartile ranges. Sample sizes: 40–49 (n = 183), 50–59 (n = 310), 60–69 (n = 347), 70–79 (n = 190), and 80+ (n = 87).

Fig. 1 illustrates the distribution of prostate volume by age group. It shows a progressive increase in volume with advancing age, consistent with the multivariate model's findings.

## Discussion

In this observational study of 1117 men, statin use ‒ defined according to electronic prescription records available in routine clinical data ‒ showed no significant association with prostate enlargement (≥ 40 mL). Likewise, no relevant correlations were found between lipid fractions (HDL, LDL, triglycerides, and total cholesterol) and prostate volume, even after adjustment for age, hypertension, and metabolic syndrome. Age was the only variable independently associated with PE, reaffirming its central physiological role in prostatic growth during the aging process.^3^^,^^9^

Although age and MetS share biological pathways, collinearity diagnostics showed only weak correlations (|*r*| < 0.20) and low VIF values (< 2.0), indicating that the loss of MetS significance after adjustment is consistent with confounding by age rather than a statistical artifact. Sensitivity analyses consistently supported these findings: the absence of association between lipids, statins, and prostate enlargement persisted in non-statin users, IPTW-weighted models, and LDL-stratified analyses. Additionally, lipid-variance testing showed lower dispersion among statin users, confirming metabolic homogenization (i.e., narrowing of lipid variability due to widespread lipid-lowering therapy and cardiometabolic management), though this did not alter the overall pattern of results.

To further clarify whether the crude association observed between metabolic syndrome and prostate enlargement reflected mediation rather than confounding, the authors conducted an exploratory mediation analysis (reported in the Supplementary Appendix). Neither model demonstrated a significant mediation effect: metabolic syndrome did not exert its effect on prostate enlargement through age, nor did age act through metabolic syndrome. In both models, the direct effect of age remained significant, while mediation effects were null. These findings reinforce the interpretation that the crude association between metabolic syndrome and prostate enlargement is primarily explained by age-related confounding with no evidence of a meaningful mediated pathway in this dataset, particularly in metabolically controlled clinical populations. These findings contrast with the existing literature, which implicates dyslipidemia in the etiology of prostatic growth.

Taken together, these results support an age-confounded interpretation of the crude MetS signal and frame the discussion of discrepant findings in prior studies.

Some studies, such as those by Besiroglu et al., found a positive association between the triglyceride/HDL ratio and prostate volume in non-diabetic patients.^7^^,^^10^^,^^13^, ^14^, ^15^, ^16^

Others have attributed antiproliferative effects to statins through inhibition of the mevalonate pathway, reduced oxidative stress, and suppression of subclinical prostatic inflammation.^5^^,^^6^^,^^17^, ^18^, ^19^ However, studies by Parsons et al., Gupta et al., and Ploumidiou et al. did not confirm these associations, which align with the present results.^4^^,^^8^^,^^9^^,^^13^

The consistency of these findings across all sensitivity analyses supports their robustness. In the non-statin subgroup, among IPTW-weighted models, and across LDL strata, age remained the only independent determinant of prostate enlargement.

Although this simplified classification limits pharmacological granularity, it reflects real-world prescription data and provides a pragmatic estimate of exposure in routine clinical practice. Future studies capturing statin type, dose, duration, and adherence are needed to evaluate potential dose-response and cumulative effects.

The observed neutrality likely reflects the clinical and metabolic characteristics of the studied cohort: over one-third of individuals were on statin therapy, and lipid values were well controlled. The statistically significant reduction in lipid parameter variability indicates that widespread treatment may homogenize metabolic profiles, limiting the detection of subtle associations. Therefore, although “treated dyslipidemia” is not formally recognized, it may be helpful for contextualizing clinical analyses in cardiometabolically monitored populations.^18^^,^^19^

Additionally, metabolic syndrome was more prevalent among patients with enlarged prostates (34.5% vs. 26.4%), but lost significance after adjustment for age. Given the low intercorrelation coefficients and VIFs, this loss of association is consistent with confounding by age rather than harmful collinearity, a pattern also reported in previous studies.^4^^,^^5^^,^^15^

PE arises from a complex interplay of hormonal, molecular, and genetic mechanisms. Among these, Dihydrotestosterone (DHT) remains the principal hormonal driver, promoting the proliferation of both stromal and epithelial cells within the transitional zone of the prostate.^20^ This process is primarily mediated by the enzyme 5-alpha-reductase, especially its type 2 isoform, which is encoded by the SRD5A2 gene.^21^^,^^22^ Genetic polymorphisms in SRD5A2 can influence local DHT production, thereby contributing to individual variability in prostate growth. Beyond hormonal regulation, inflammatory gene polymorphisms ‒ including those in IL-6, IL-8, and TNF-α ‒ may also affect the rate and extent of prostatic enlargement, independent of metabolic status.^23^^,^^24^

Although PE is typically associated with aging and benign prostatic changes, some studies suggest that tiny prostate volumes may also have clinical implications. Chronic ischemia, resulting from reduced pelvic perfusion, has been linked to decreased prostate size due to fibrosis and glandular atrophy.^25^^,^^26^ In such scenarios, Lower Urinary Tract Symptoms (LUTS) may arise more from bladder dysfunction than from prostate-related obstruction. These findings raise the possibility of a bimodal relationship between vascular health and prostate volume, in which both excessive growth and ischemic shrinkage can negatively affect urinary function via distinct pathophysiological pathways.^26^^,^^27^

It is also important to acknowledge that the present analysis focused exclusively on prostate volume as a morphological surrogate of BPH, without evaluating lower urinary tract symptoms or functional outcomes. This distinction matters, as prostate enlargement and LUTS are not always concordant, and prior studies have suggested that metabolic factors may have a stronger association with LUTS than with prostate size per se.^28^, ^29^, ^30^ For instance, components of metabolic syndrome ‒ such as insulin resistance, abdominal obesity, and systemic inflammation ‒ have been linked to bladder dysfunction, detrusor overactivity, and impaired relaxation of the prostatic urethra, all of which can contribute to LUTS independent of prostate volume.^31^ Thus, the lack of association between lipid profile, statin use, and prostate enlargement observed in the present study does not exclude a potential pathophysiological or clinical relevance of these factors in the etiology or exacerbation of LUTS.

The present findings should not be extrapolated to symptomatic BPH or metabolically uncontrolled populations. In a cohort with a high prevalence of statin therapy and narrow lipid dispersion, prostate enlargement appears predominantly age-related.

Future studies that incorporate both anatomical measures and patient-reported symptoms are needed to clarify the overlapping yet distinct contributions of metabolic health to the structural and functional components of male lower urinary tract dysfunction.

This study has limitations, including its cross-sectional design, which limits causal inference; the absence of detailed data on statin type, dose, duration, and adherence; the lack of hormonal (free testosterone, DHT) and inflammatory (CRP, IL-6, TNF-α) biomarkers; and the use of prostate volume as a morphological surrogate rather than a full clinical definition that includes symptoms or functional assessment. Although all ultrasounds were performed by the same radiology team using a standardized protocol, inter-observer variability was not formally assessed.

However, this work has notable strengths: a large sample size, the use of routine clinical data, standardized ultrasound measurements, and multivariable adjustment for confounders. The inclusion of multiple sensitivity analyses, formal collinearity testing, and variance assessments considerably strengthens the robustness of interpretation. The use of metabolic syndrome as a composite construct, rather than its isolated components, enhances interpretive robustness. Future studies should incorporate genetic, hormonal, and inflammatory biomarkers, as well as comprehensive medication data and longitudinal designs, to gain a deeper understanding of this multifaceted interaction.

## Conclusions

Age emerged as the only variable independently associated with prostate enlargement, reaffirming its central role in prostatic growth during the aging process. In contrast, a prescription-based measure of statin use, lipid parameters (HDL, LDL, triglycerides, and total cholesterol), and metabolic syndrome showed no independent associations after adjustment, indicating that the modest crude associations observed with metabolic components are explained by age and are consistent with confounding by age; exploratory mediation analyses did not provide evidence of a meaningful mediated pathway in this dataset.

In this cohort of metabolically well-controlled men ‒ with a high prevalence of statin therapy and narrow lipid variability ‒ no evidence was found that modifiable metabolic factors influence prostate volume once age is accounted for. These findings are consistent with prostate enlargement being predominantly age-related in this clinical setting, rather than primarily driven by metabolic dysregulation.

These results should not be extrapolated to symptomatic BPH or metabolically uncontrolled populations. Future longitudinal and mechanistic studies integrating genetic, hormonal, and inflammatory biomarkers, as well as detailed statin exposure data (type, dose, duration, and adherence), are warranted to clarify how these pathways interact with aging in determining prostatic growth and to identify potential subgroups that may benefit from metabolic or pharmacologic modulation.

## Human ethics

The following information was supplied relating to ethical approvals (i.e., approving body and any reference numbers): this project was approved by the Research Ethics Committee of the State University of Feira de Santana (protocol n° 97,443,018.3.0000.0053, position statement 3.057.301, on December 5, 2018). The present analysis uses data collected between 2021 and 2023.

## Funding

The authors received no funding for this work.

## Data availability statement

The datasets generated and/or analyzed during the current study are available from the corresponding author upon reasonable request.

## CRediT authorship contribution statement

**Lucas Neves de Oliveira:** Conceptualization, Methodology, Investigation, Formal analysis, Visualization, Writing – original draft, Writing – review & editing. **Ricardo Brianezi Tiraboschi:** Conceptualization, Methodology, Investigation, Formal analysis, Writing – review & editing. **Caroline Santos Silva:** Conceptualization, Methodology, Investigation, Formal analysis, Writing – review & editing. **Jair Bomfim Santos:** Conceptualization, Methodology, Investigation, Formal analysis, Writing – review & editing. **Mateus Neves de Oliveira:** Conceptualization, Methodology, Investigation, Formal analysis, Writing – review & editing. **Cristiano Mendes Gomes:** Conceptualization, Formal analysis, Visualization, Supervision, Writing – review & editing. **Jose Bessa Junior:** Conceptualization, Methodology, Investigation, Formal analysis, Visualization, Supervision, Writing – review & editing.

## Declaration of competing interest

The authors declare no conflicts of interest.

## Appendix Supplementary materials

Image, application 1

Image, application 2
