# Supplementary material for: Age trumps metabolism: No independent association between lipids, statins, and prostate enlargement in a metabolically controlled cohort
Source: Clinics (Sao Paulo). 2026 May 20;81:100939. doi: 10.1016/j.clinsp.2026.100939 (PMC13214303; doi:10.1016/j.clinsp.2026.100939)
Supplement: Supplementary file 1 [file mmc1.docx]

**CLINICS-D-25-01582**

**Supplementary Appendix**

**Table S1** Restricted analysis in non-users of statins (logistic regression; outcome: PE [≥ 40 mL]).

| **Covariate** | **OR** | **95% CI (low–high)** | **p-value** |
| --- | --- | --- | --- |
| Age (per year) | 1.06 | 1.04‒1.07 | <0.001 |
| HTN (yes) | 1.14 | 0.80‒1.62 | 0.5 |
| Waist > 102 cm (yes) | 0.94 | 0.64‒1.37 | 0.7 |
| LDL (per mg/dL) | 1.00 | 1.00‒1.01 | 0.6 |
| HDL (per mg/dL) | 1.00 | 0.99‒1.00 | 0.5 |
| Triglycerides (per mg/dL) | 1.00 | 1.00‒1.00 | >0.9 |
| Glycemia (per mg/dL) | 1.00 | 1.00‒1.01 | 0.2 |

OR, Odds Ratio; CI, Confidence Interval, HTN, Hypertension.

Model: PE ~ age + hypertension + waist > 102 + LDL + HDL + Triglycerides + glycemia.

**Table S2** PS-IPTW outcome model (weights from Statin ~ age + HTN + LDL + HDL + TG + waist > 102 + glycemia).

| **Covariate** | **OR** | **95% CI (low–high)** | **p-value** |
| --- | --- | --- | --- |
| Statin user (yes) | 0.94 | 0.73‒1.21 | 0.6 |
| Age (per year) | 1.05 | 1.04‒1.06 | <0.001 |

OR, Odds Ratio; CI, Confidence Interval; GLM, Binomial with stabilized IPTW.

**Table S3** Stratified models by lipid control.

| **A** ‒ LDL <100 mg/dL | | | | | |
| --- | --- | --- | --- | --- | --- |
| **Covariate** | **OR** | **95% CI (low–high)** | | **p-value** | |
| Age (per year) | 1.04 | 1.02‒1.06 | | <0.001 | |
| Statin user (yes) | 1.44 | 0.94‒2.19 | | 0.092 | |
| HTN (yes) | 0.94 | 0.61‒1.46 | | 0.8 | |
| Waist > 102 cm (yes) | 1.40 | 0.91‒2.16 | | 0.12 | |
| **B** ‒ LDL ≥100 mg/dL | | | | | |
| **Covariate** | **OR** | | **95% CI (low–high)** | | **p-value** |
| Age (per year) | 1.07 | | 1.05‒1.09 | | <0.001 |
| Statin user (yes) | 0.74 | | 0.50‒1.10 | | 0.14 |
| HTN (yes) | 1.36 | | 0.95‒1.94 | | 0.092 |
| Waist > 102 cm (yes) | 1.12 | | 0.77‒1.61 | | 0.5 |

OR, Odds Ratio; CI, Confidence Interval; HTN, Hypertension.

**Table S4** Variance tests for lipid fractions by statin status.

| **Marker** | **N (users)** | **N (non users)** | **SD (users)** | **SD (non users)** | **Levene p** | **Fligner p** |
| --- | --- | --- | --- | --- | --- | --- |
| LDL | 410 | 707 | 34.42 | 63.32 | 0.033 | 0.052 |
| HDL | 410 | 707 | 11.46 | 24.09 | 0.338 | 0.656 |
| TG | 410 | 707 | 82.16 | 96.10 | 0.011 | 0.002 |

**Table S5** Mediation analysis results for prostate enlargement (≥40 mL).

| **Analysis Model** | **Path Type** | **Estimate** | **95% Confidence Interval** | **p-value** |
| --- | --- | --- | --- | --- |
| Model A | Indirect Effect (ACME) | 0.0145 | [-0.0023, 0.03] | 0.082 |
| (X: MetS → M: Age → Y: PE) | Direct Effect (ADE) | 0.0295 | [-0.0281, 0.09] | 0.334 |
|  | Total Effect | 0.0440 | [-0.0167, 0.11] | 0.148 |
| Model B | Indirect Effect (ACME) | 0.00003 | [-0.0001, 0.0001] | 0.940 |
| (X: Age → M: MetS → Y: PE) | Direct Effect (ADE) | 0.0011 | [0.0006, 0.0015] | <0.001 |
|  | Total Effect | 0.0011 | [0.0006, 0.0016] | <0.001 |

Effects estimated on the model scale (as returned by the mediation package).
