# Supplementary material for: Age trumps metabolism: No independent association between lipids, statins, and prostate enlargement in a metabolically controlled cohort
Source: Clinics (Sao Paulo). 2026 May 20;81:100939. doi: 10.1016/j.clinsp.2026.100939 (PMC13214303; doi:10.1016/j.clinsp.2026.100939)
Supplement: Supplementary file 2 [file mmc2.docx]

Supplementary Appendix

**Table S1**. Restricted analysis in non-users of statins (logistic regression; outcome: PE(≥40 mL)

| **Covariate** | **OR^1^** | **95% CI^1^** (low–high) | **p-value** |
| --- | --- | --- | --- |
| Age (per year) | 1.06 | 1.04 - 1.07 | <0.001 |
| HTN (yes) | 1.14 | 0.80 - 1.62 | 0.5 |
| Waist > 102 cm (yes) | 0.94 | 0.64 - 1.37 | 0.7 |
| LDL (per mg/dL) | 1.00 | 1.00 - 1.01 | 0.6 |
| HDL (per mg/dL) | 1.00 | 0.99 - 1.00 | 0.5 |
| Triglycerides (per mg/dL) | 1.00 | 1.00 - 1.00 | >0.9 |
| Glycemia (per mg/dL) | 1.00 | 1.00 - 1.01 | 0.2 |
| ^1^OR = Odds Ratio, CI = Confidence Interval, HTN = hypertension. | | | |

Model: PE ~ age + hypertension + waist>102 + LDL + HDL + Triglycerides + glycemia.

**Table S2.** PS-IPTW outcome model

(weights from Statin~age+HTN+LDL+HDL+TG+waist>102+glycemia)

| **Covariate** | **OR^1^** | **95% CI^1^ (low–high)** | **p-value** |
| --- | --- | --- | --- |
| Statin user (yes) | 0.94 | 0.73 - 1.21 | 0.6 |
| Age (per year) | 1.05 | 1.04 - 1.06 | <0.001 |
| ^1^ OR = Odds Ratio, CI = Confidence Interval | | | |

GLM binomial with stabilized IPTW

**Table S3.** Stratified models by lipid control

A. LDL <100 mg/dL

| **Covariate** | **OR^1^** | **95% CI^1^(low–high)** | **p-value** |
| --- | --- | --- | --- |
| Age (per year) | 1.04 | 1.02 - 1.06 | <0.001 |
| Statin user (yes) | 1.44 | 0.94 - 2.19 | 0.092 |
| HTN (yes) | 0.94 | 0.61 - 1.46 | 0.8 |
| Waist > 102 cm (yes) | 1.40 | 0.91 - 2.16 | 0.12 |
| ^1^ OR = Odds Ratio, CI = Confidence Interval, HTN = hypertension. | | | |

B. LDL ≥100 mg/dL

| **Covariate** | **OR^1^** | **95% CI^1^ (low–high)** | **p-value** |
| --- | --- | --- | --- |
| Age (per year) | 1.07 | 1.05 - 1.09 | <0.001 |
| Statin user (yes) | 0.74 | 0.50 - 1.10 | 0.14 |
| HTN (yes) | 1.36 | 0.95 - 1.94 | 0.092 |
| Waist > 102 cm (yes) | 1.12 | 0.77 - 1.61 | 0.5 |
| ^1^ OR = Odds Ratio, CI = Confidence Interval, HTN = hypertension. | | | |

**Table S4.** Variance tests for lipid fractions by statin status

| **Marker** | **N**  **(users)** | **N**  **(non users)** | **SD (users)** | **SD**  **(non users)** | **Levene p** | **Fligner p** |
| --- | --- | --- | --- | --- | --- | --- |
| LDL | 410 | 707 | 34,42 | 63.32 | 0.033 | 0.052 |
| HDL | 410 | 707 | 11.46 | 24.09 | 0.338 | 0.656 |
| TG | 410 | 707 | 82.16 | 96.10 | 0.011 | 0.002 |

**Table S5.** Mediation Analysis Results for Prostate Enlargement (≥40 mL)

| Analysis Model | Path Type | Estimate | 95% Confidence Interval | p-value |
| --- | --- | --- | --- | --- |
| Model A | Indirect Effect (ACME) | 0.0145 | [-0.0023, 0.03] | 0.082 |
| (X: MetS → M: Age → Y: PE) | Direct Effect (ADE) | 0.0295 | [-0.0281, 0.09] | 0.334 |
|  | Total Effect | 0.0440 | [-0.0167, 0.11] | 0.148 |
|  |  |  |  |  |
| Model B | Indirect Effect (ACME) | 0.00003 | [-0.0001, 0.0001] | 0.940 |
| (X: Age → M: MetS → Y: PE) | Direct Effect (ADE) | 0.0011 | [0.0006, 0.0015] | < 0.001 |
|  | Total Effect | 0.0011 | [0.0006, 0.0016] | < 0.001 |

Effects estimated on the model scale (as returned by the mediation package)”
